# A case of percutaneous transhepatic stomal varices embolization and partial splenic artery embolization for rectal cancer after CAPOX/BEV chemotherapy: the summary of the stomal varices related to oxaliplatin administration

**DOI:** 10.1007/s12328-022-01720-7

**Published:** 2022-10-20

**Authors:** Shoichiro Mizukami, Tatsuya Shonaka, Chikayoshi Tani, Kazuki Ihara, Tomohiro Takeda, Mizuho Ohara, Kimiharu Hasegawa, Mishie Tanino, Koji Sawada, Yasuo Sumi

**Affiliations:** 1 Division of Gastrointestinal Surgery, Department of Surgery, Asahikawa Medical University, 2-1-1-1 Midorigaoka-Higashi, Asahikawa, Hokkaido 078-8510 Japan; 2 Department of Diagnostic Pathology, Asahikawa Medical University, 1-1-1 Midorigaoka-Higashi, Asahikawa, Hokkaido 078-8510 Japan; 3 Division of Metabolism and Biosystemic Science, Gastroenterology, and Hematology/Oncology, Department of Medicine, Asahikawa Medical University, 2-1-1-1 Midorigaoka-Higashi, Asahikawa, Hokkaido 078-8510 Japan

**Keywords:** Stomal varices, Sinusoidal obstruction syndrome, Oxaliplatin

## Abstract

Capecitabine and oxaliplatin (CAPOX) plus bevacizumab (BEV) therapy (CAPOX/BEV) is a standard treatment recommended as the first-line treatment for colorectal cancer recurrence. Recently, sinusoidal obstruction syndrome (SOS) and resulting portal hypertension have been reported as important side effects of oxaliplatin. We herein report a rectal cancer patient who underwent percutaneous transhepatic stoma variceal embolization (PTO) and partial splenic artery embolization (PSE) for stomal variceal bleeding and splenomegaly due to portal hypertension caused by SOS after CAPOX therapy. A 43-year-old man who underwent robot-assisted laparoscopic abdominoperineal resection for advanced lower rectal cancer was started on CAPOX/BEV therapy for early recurrence 1 month after surgery. In the sixth course, splenomegaly rapidly worsened, stomal varices appeared, and the stoma began bleeding. At 5 months after the appearance of stomal varices, the splenomegaly worsened, the frequency of stomal bleeding increased, and PTO was performed. Five months later, PSE was performed for splenomegaly and thrombocytopenia. At 5 months since the PSE, the stoma bleeding has not recurred, and the thrombocytopenia has been corrected. The patient has been able to continue chemotherapy. We suggest that staged treatment by PTO and PSE be considered an important treatment option for stomal varices and splenomegaly associated with SOS.

## Introduction

CAPOX therapy is a standard treatment recommended as adjuvant chemotherapy and primary treatment for recurrence of colorectal cancer, but sinusoidal obstruction syndrome (SOS) has been reported as an important side effect of oxaliplatin [1, 2]. SOS reportedly causes fibrosis and stenosis of sinusoids, which eventually leads to portal hypertension [3, 4], and portal hypertension increases inferior mesenteric vein (IMV) pressure, resulting in the appearance of stoma varicose veins [5].

We herein report a case of rectal cancer in which percutaneous transhepatic stoma variceal embolization (PTO) and partial splenic artery embolization (PSE) were performed due to stomal varices caused by portal hypertension induced by SOS after oxaliplatin administration.

## Case report

**Fig. 1:**
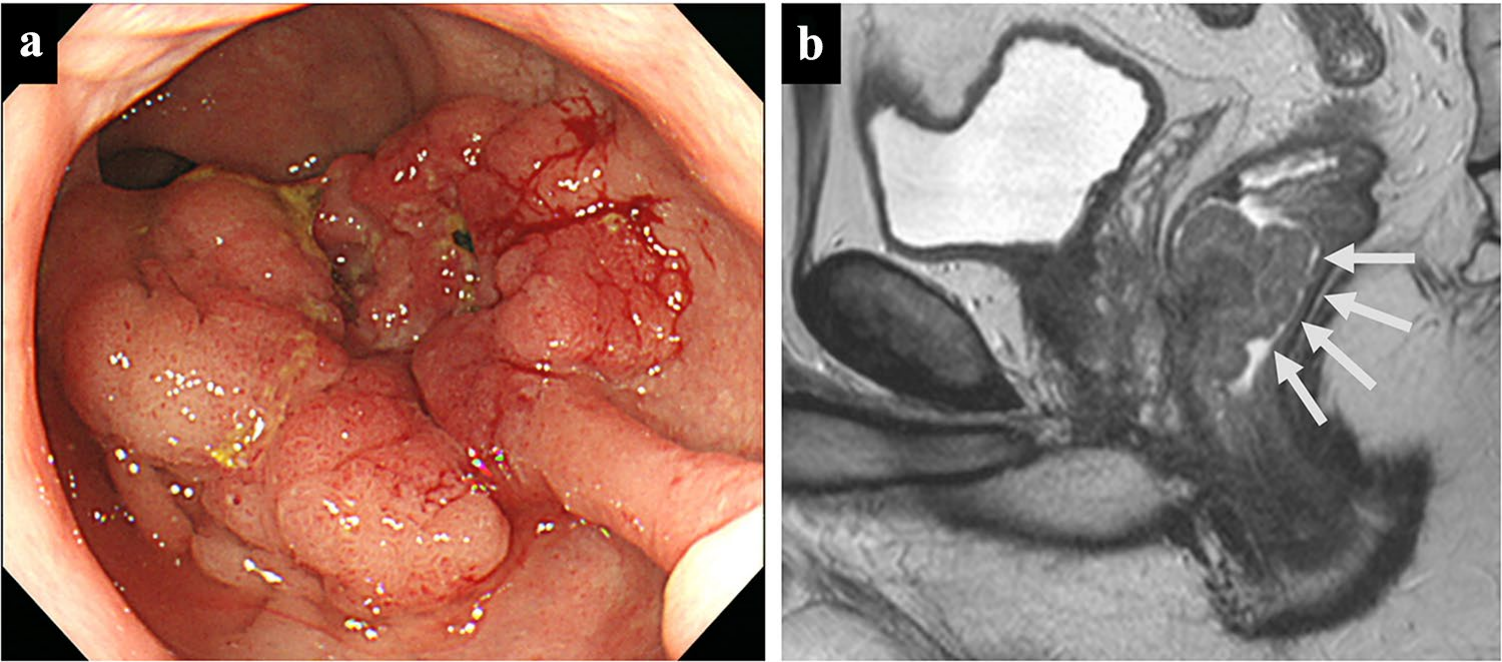
Colonoscopy and MRI at before the surgical treatment of rectal cancer. **a** Colonoscopy revealed a half circular 40-mm type 2 tumor in the anterior wall of the lower rectum. **b** MRI (T2WI: sagittal) revealed an irregular mass on the anterior wall side of the lower rectum; prostate invasion was suspected (arrows)

A 43-year-old man was diagnosed with advanced lower rectal cancer (Rb, cT4b, cN3, cM0, cStage IIIc [6]) (Fig. 1a, b) and underwent robot-assisted laparoscopic abdominoperineal resection with bilateral lymph node dissection. He had no remarkable personal or family history.

**Fig. 2:**
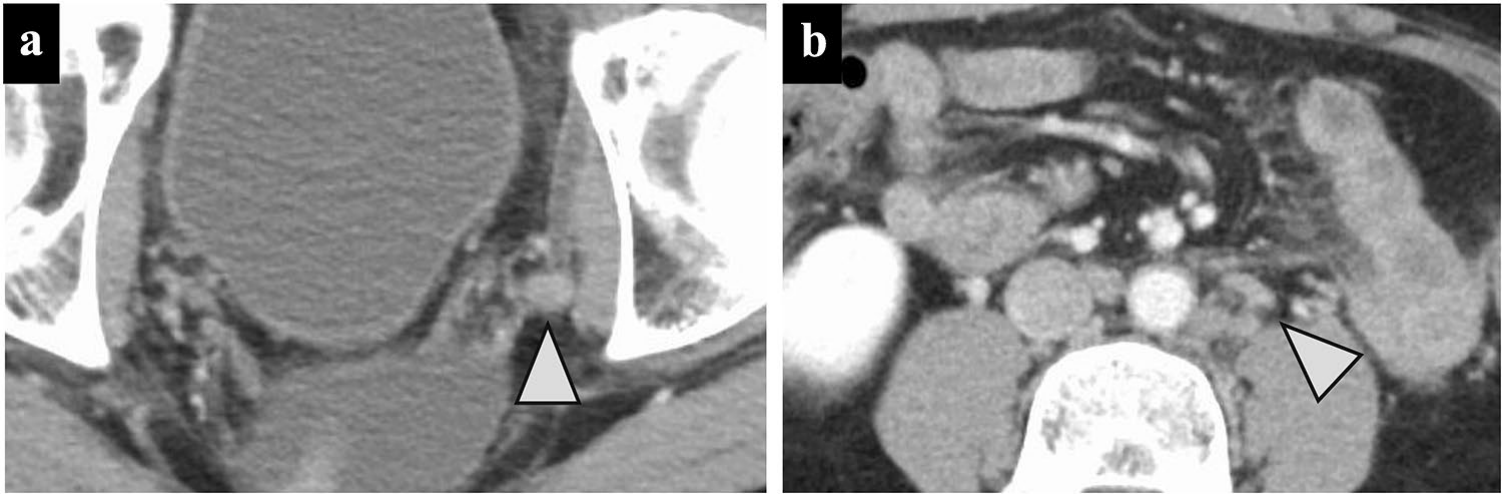
Contrast-enhanced CT findings at the time of early recurrence at the lymph nodes. **a** The metastatic left lateral lymph node was enlarged (arrowhead). **b** The para-aortic lymph nodes were enlarged (arrowhead)

One month postoperatively, contrast-enhanced computed tomography (CT) revealed multiple enlarged left lateral lymph nodes and para-aortic lymph nodes (Fig. 2a, b), leading to the diagnosis of early recurrence, and CAPOX/BEV therapy (arm: bevacizumab 7.5 mg/kg and oxaliplatin 130 mg/m^2^ on day 1 and oral capecitabine 2000 mg/m^2^ on days 1–14, every 3 weeks) was initiated. The splenomegaly worsened rapidly after the sixth course of CAPOX, and the platelet count decreased. He had no abdominal pain suspicious of hepatomegaly and no weight gain.

Five months after starting chemotherapy, the patient developed stomal varices and began bleeding (approximately 300–500 ml each time). After nine courses of CAPOX/BEV had been administered, the neuropathy worsened, and oxaliplatin was discontinued (total dose of oxaliplatin: 1084 mg/m^2^). Thereafter, contrast-enhanced CT determined he had progressive disease (PD) [7], and two courses of FOLFIRI plus Aflibercept therapy were administered.

Thirteen months after starting chemotherapy, contrast-enhanced CT revealed worsening of the splenomegaly and stomal varices as well as frequent bleeding from the stoma, so the patient was admitted to the hospital for a close examination and treatment. Hematological and biochemical examinations on admission revealed a slightly low level of hemoglobin 11.0 g/dl and a platelet count of 15.2 × 10^4^/mm^3^. The coagulation function and hepatobiliary enzyme levels were normal. The liver function was acceptable (Table 1).

**Table 1:** Laboratory data on admission

Blood count		Biochemistry		Infection		
WBC	5840/mm^3^	Alb	3.8 g/dL	HBs Ag	< 0.01 IU/mL	
Hgb	11.0 g/dl	T.Bil	0.6 mg/dL	HBs Ab	< 2.5 mIU/mL	
Plt	15.2 × 10^4^/mm^3^	D.Bil	0.1 mg/dL	HCV Ab	0.05 S/CO	
Coagulation		AST	25 U/L	Tumor marker		
		ALT	18 U/L			
PT%	111%	LDH	168 U/L	CEA	13.5 ng/ml	
PT INR	0.92	ALP	211 U/L	CA19-9	72 U/ml	
APTT	27.5 s	γGT	36 U/L	Liver function		
Fib	297 μg/mL	BUN	14.8 mg/dL			
FDP	3.6 μg/mL	Cre	0.8 mg/dL	Child Pugh score		5 (A)
D.D	1.9 μg/mL	CRP	< 0.1 mg/dL	ALBI score		− 2.56
AT-III	116%	NH_3_	82 µg/dL	MELD score		1

**Fig. 3:**
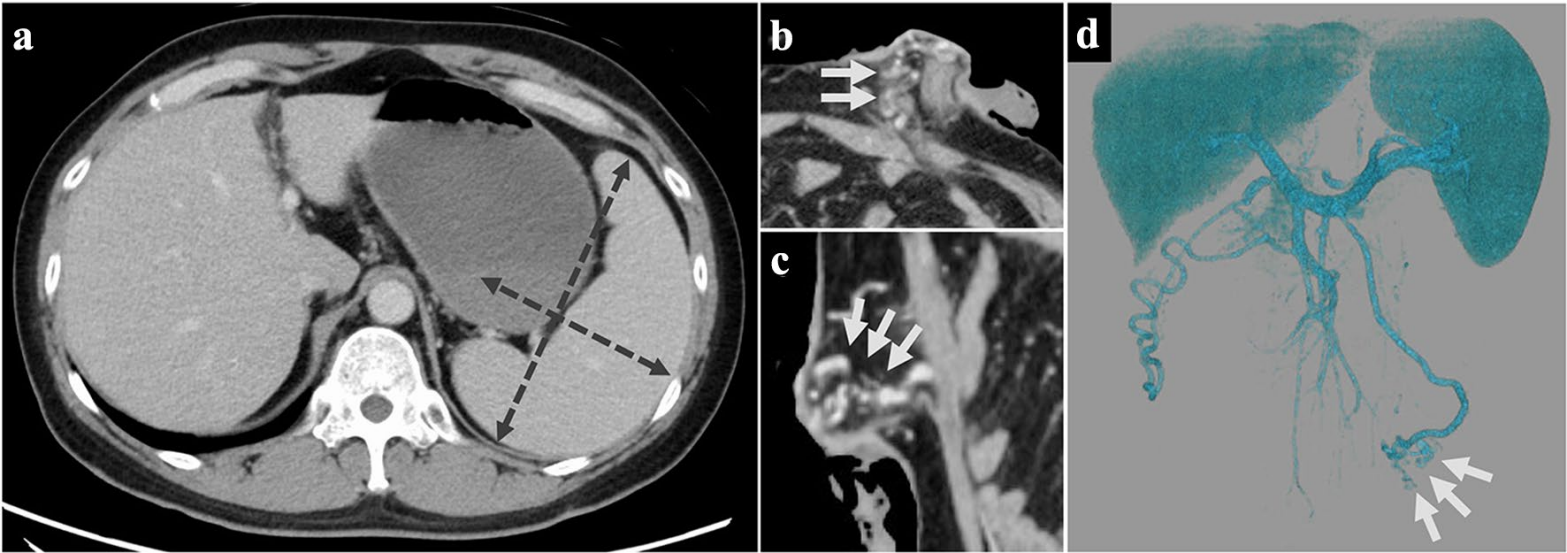
Contrast-enhanced CT findings at admission. **a** Severe splenomegaly 17 × 7 cm (dotted arrow). No liver metastases were found. **b**–**d** Varicose veins around the stoma (arrow) perfused into the IMV (**b** axial, **c** sagittal, **d** 3D angiography)

**Fig. 4:**
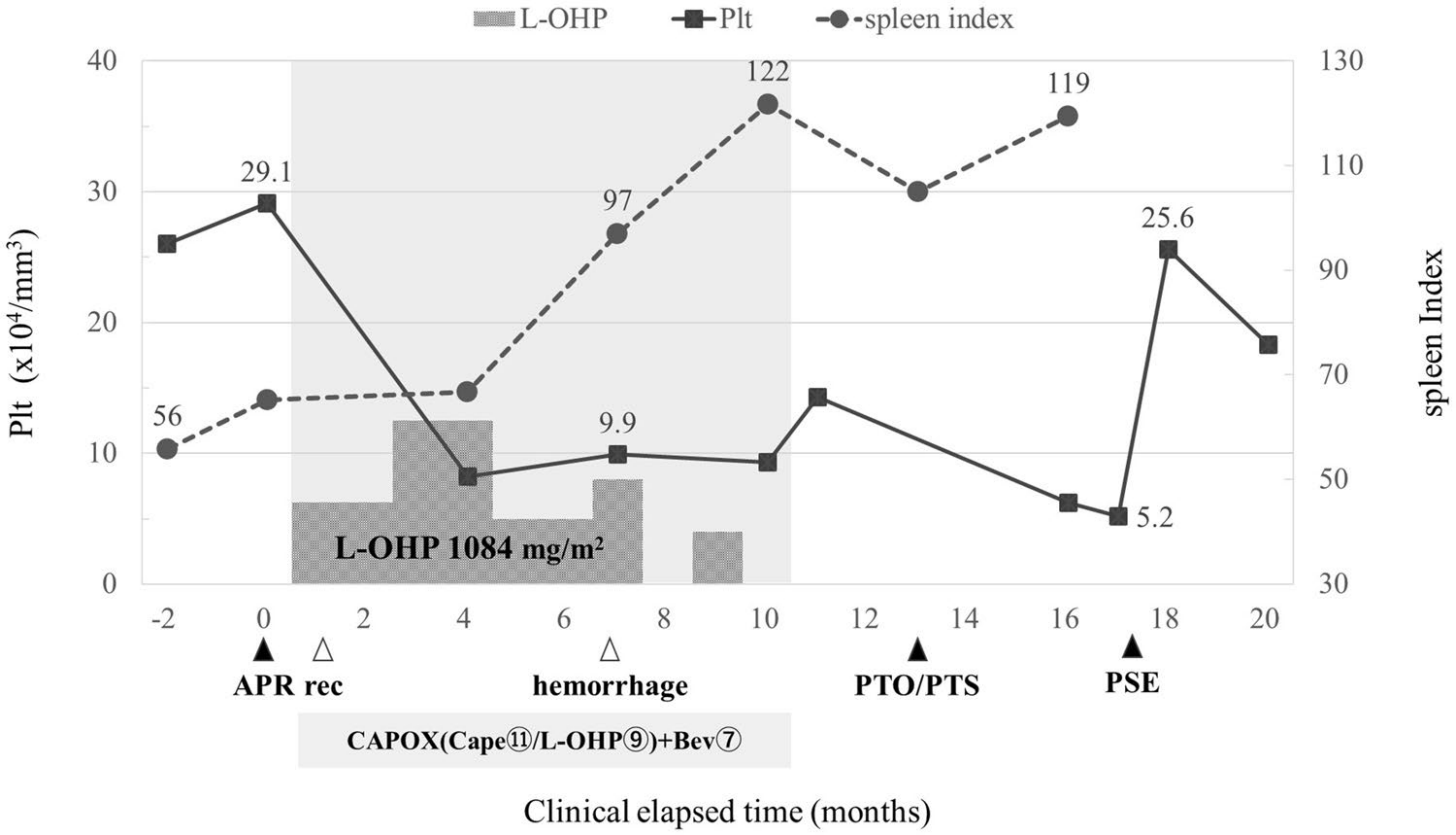
Clinical course and changes in the platelet count and spleen index

Contrast-enhanced CT findings on admission showed a 17 × 7 cm splenomegaly (Fig. 3a) and stomal varices (Fig. 3b–d). A comparison of the CT findings at the time of early recurrence with the CT findings obtained before admission showed that the spleen index (splenic length diameter × splenic width diameter) had doubled, and the stomal varices were highly developed (Fig. 4). The thickness of the liver at the midline of the clavicle was 16 cm, suggesting mild hepatomegaly. No liver metastasis findings were observed during the disease course, and no varicose veins were observed by gastrointestinal endoscopy through the stoma.

**Fig. 5:**
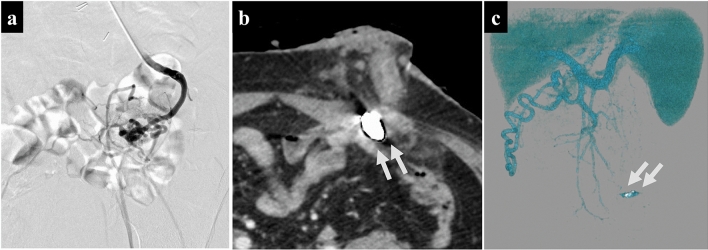
Percutaneous transhepatic obliteration. **a** Fluoroscopic angiography revealed that the peristomal varices were visualized from the inferior abdominal wall vein to the external iliac vein. **b**, **c** Postoperative contrast-enhanced CT revealed the disappearance of stomal varices (arrow) (**b** axial, **c** 3D angiography)

We decided to perform a liver biopsy and percutaneous transhepatic stomal variceal embolization to investigate SOS and treat the stomal varices. We approached the stomal varices from the right branch of portal vein and performed inferior mesenteric vein (IMV) angiography. The peristomal varices were visualized from the inferior abdominal wall vein to the external iliac vein (Fig. 5a). We injected 5% Ethanolamine oleate into the peripheral stomal varices and embolized the central side with Fibered IDC coil^®^ 5 mm × 12 cm and C-Stopper coil^®^0.016 180 mm × 3.

**Fig. 6:**
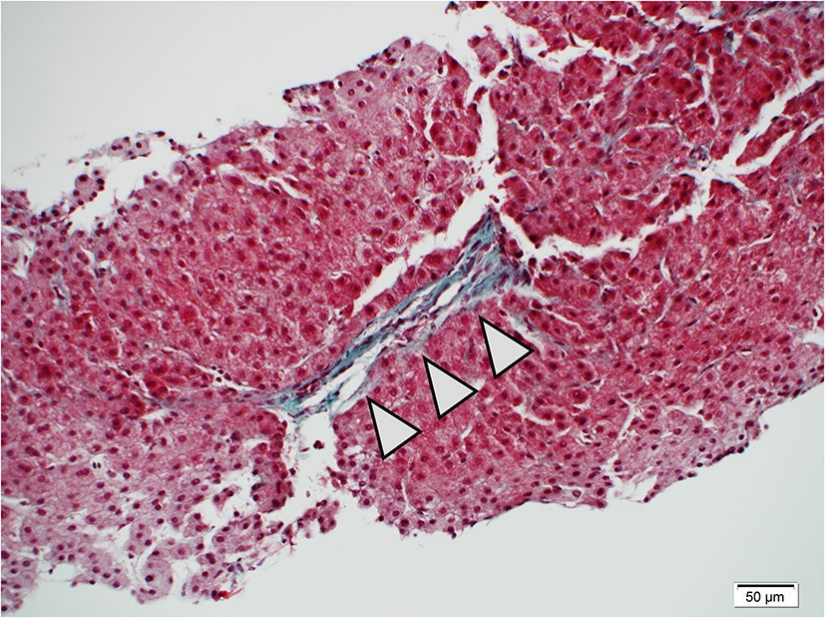
Histopathological examination findings of a liver biopsy specimen. A trichrome stain demonstrated focal central vein narrowing and sinusoidal occlusion by fibrosis (arrowhead), consistent with sinusoidal obstruction syndrome

Postoperative contrast-enhanced CT revealed the disappearance of the stomal varices (Fig. 5b, c). A histopathological examination of a liver biopsy specimen demonstrated focal central vein narrowing and sinusoidal occlusion by fibrosis, consistent with sinusoidal obstruction syndrome (Fig. 6). Based on the European Society for Blood and Marrow Transplantation (EBMT) criteria, which are currently used to diagnose and classify the severity of SOS, the severity was determined to be mild [8].

**Fig. 7:**
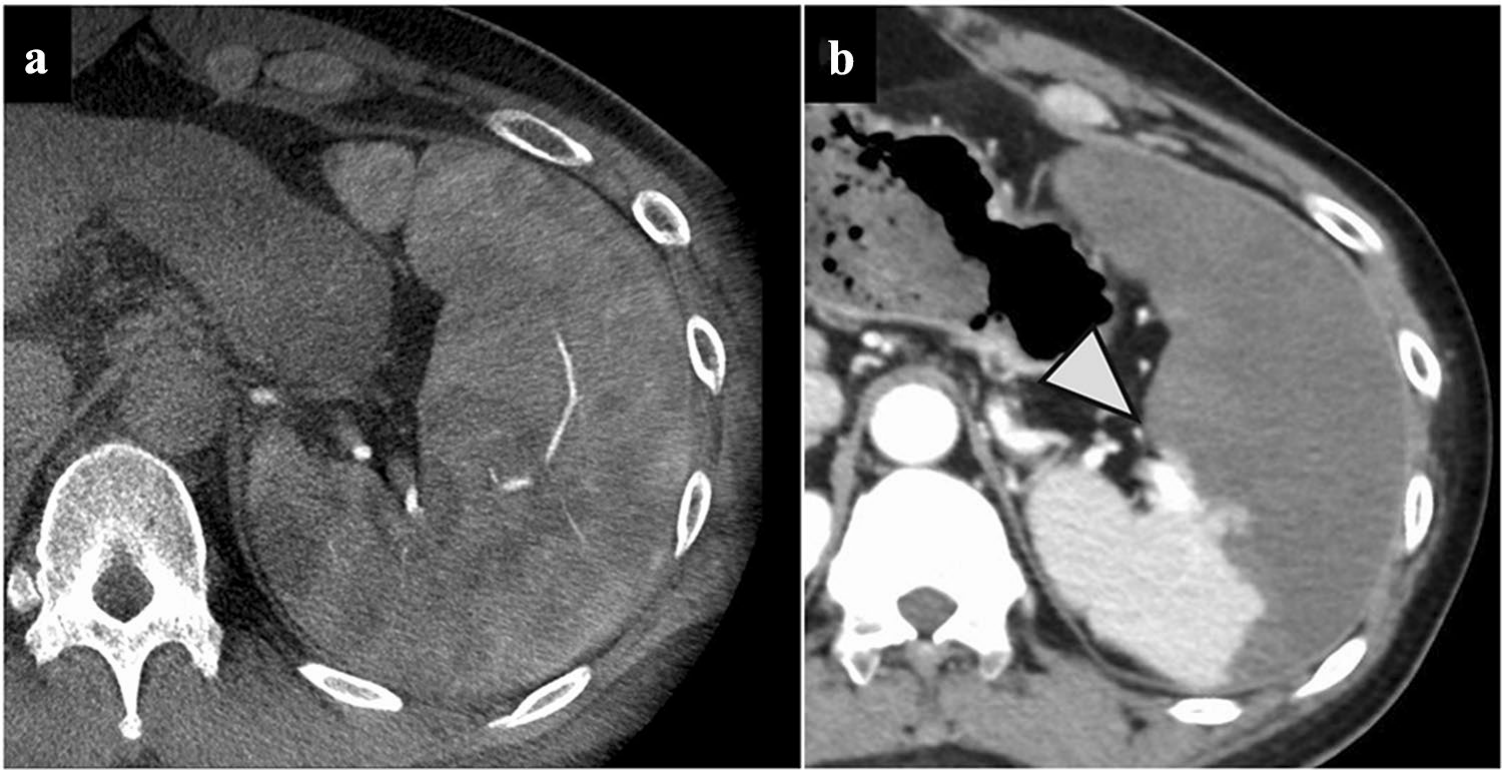
Contrast-enhanced CT during PSE. The middle to lower poles of the spleen were poorly contrasted (**a**. preoperative CT scan, **b**. postoperative CT scan; arrowhead: boundary of the infarcted spleen)

We performed PSE 5 months after PTO because the splenomegaly induced thrombocytopenia made it difficult to continue chemotherapy. We punctured the right femoral artery and selectively embolized the middle and inferior pole branches of the splenic artery with Spongel^®^. Postoperative contrast-enhanced CT showed that the middle to lower poles of the spleen were almost entirely contrast-enhanced, except for some areas, and the embolization effect was about 80% (Fig. 7a, b). The platelet count improved quickly after PSE (Fig. 4). Four months have passed after PSE, and chemotherapy is ongoing without any recurrence of stoma bleeding.

## Discussion

SOS is a systemic endothelial disease that typically presents in the days or weeks after hematopoietic cell transplantation (HCT) with refractory thrombocytopenia, hepatomegaly, ascites, and jaundice, and it can rapidly progress to multiorgan dysfunction and death. SOS develops in up to 15% of adults after HCT [9], but has also been reported as a major side effect of oxaliplatin [1, 2]. It is induced by damage to sinusoidal endothelial cells and amplified by the local inflammatory response and activation of the coagulation-fibrinolysis pathway, leading to severe hepatic necrosis [3, 4]. Regarding the underlying mechanism, oxaliplatin causes the generation of reactive oxygen species, which depletes glutathione in sinusoidal epithelial cells and increases oxidative stress [10]. In addition, oxaliplatin causes depolymerization of actin in sinusoidal epithelial cells and the upregulation of matrix metalloproteinases-2,9 which leads to fibroblast proliferation and endothelial cell loss as well as fibrosis of sinusoids and central veins, resulting in portal hypertension [1, 10–13].

The incidence of SOS after oxaliplatin administration was 48.8–59.0% [14–16], and SOS was reported to be found in 51% of cases of hepatic resection after neoadjuvant chemotherapy and 79% of liver biopsy specimens [1, 11], so there is concern about the high incidence of SOS. Previously, mortality from severe SOS has been reported to exceed 80% [9, 17–19]. In a systematic review of patients with severe SOS after hepatectomy, an increased frequency of postoperative complication rates, complications specific to liver surgery and postoperative liver failure were reported [20]. As for the relationship between the dose of oxaliplatin and the development of SOS, it has been reported that more careful follow-up is important in patients who have received more than 6 courses of oxaliplatin-based chemotherapy [21, 22]. It has been reported that splenomegaly is induced when the total oxaliplatin dose exceeds 1040 mg/mm^2^ and that 12 courses of FOLFOX therapy and more than 8 courses of CAPOX therapy are risk factors for the development of SOS [23]. Welsh et al. reported that postoperative complications, the reoperation rate, and the duration of hospital stay were increased in cases of hepatic resection performed after oxaliplatin administration when chemotherapy was administered for more than 12 weeks or when the time between chemotherapy discontinuation and resection was less than 4 weeks [24]. In the present case, splenomegaly worsened after the sixth course of CAPOX (oxaliplatin dose: 684 mg/m^2^), and stoma bleeding occurred in the eighth course. The platelet count decreased with the worsening of splenomegaly, and a liver biopsy at the time of stoma variceal embolization showed scattered fibrosis between hepatocytes and partial strong fibrosis in the central vein, which led to the diagnosis of SOS and portal hypertension.

Stomal varices occur at the border between the intestine and abdominal wall at the stoma-mucosal junction as a blood channel of the portal-systemic shunt under conditions of portal hypertension [5]. A total of 27–31% of patients with portal hypertension due to chronic liver disease reportedly develop varicose veins when colostomy is performed [25].

Since they form on or near the body surface, the site of bleeding is often easy to identify, and the bleeding-related mortality rate is as low as 3–4% [26]. However, bleeding stomal varices present with frequent bleeding and are often difficult to treat [27, 28]. In patients with metastatic multiple liver tumors and ascites, treatment options are limited. Bleeding due to varices is a complication that not only decreases the patient’s quality of life but also leads to progressive anemia, a worsened nutritional status, and a decreased performance status, making interruption of chemotherapy a prognostic factor. Management of stomal varices bleeding and thrombocytopenia due to splenomegaly has a significant impact on the quality of life and continuation of chemotherapy.

The two major elements of treatment are bleeding control of stoma varices and modification of the macrocirculation to improve portal hypertension. Local therapies for hemostasis of stoma varicose veins include compression, suturing, and sclerotherapy, but all are symptomatic, and rebleeding is inevitable. Drug therapies, such as β-blockers, octreotide, and ursodeoxycholic acid, are sometimes used prophylactically, but the efficacy of monotherapy is very limited [29, 30]. Stoma reconstruction is reported to be highly invasive and rebleeding occurs in 75% of cases [31]. Therefore, therapeutic intervention for the feeding vein is necessary. In our case, we chose the PTO approach from the feeding vein.

PTO is a feasible and safe option for stomal variceal bleeding, despite the invasiveness of transhepatic puncture [32–34]. Ishikawa et al. performed PTO in 37 patients with refractory hepatic encephalopathy and gastroesophageal varices and reported that the procedure was successful in all patients, with a recurrence rate of 2.7% [35]. Maciel et al. also reported that stoma variceal embolization with PTO was performed, and the bleeding subsided after 6 months of follow-up [36]. Compared with the method of percutaneous puncture and contrast of varicose veins, the overall image, including varicose veins and collateral vessels, can be grasped, which enables more efficient hemostasis by embolization [36, 37]. In the present case, we performed PTO for bleeding control. Although minimally invasive, PTO provides valuable information based on the findings of a blood flow evaluation and liver biopsy and the response rate is reported to be high. We, therefore, believe that PTO can be an important local treatment option.

After embolization of the stomal varices in our patient, the thrombocytopenia caused by splenomegaly worsened and chemotherapy could not be continued. We, therefore, also needed to address the portal hypertension. Splenectomy [38, 39], PSE [40], transjugular intrahepatic portosystemic shunt (TIPS) [3], and surgical shunt angioplasty have been reported as treatments to reduce portal pressure. PSE is useful as a method to correct abnormal splenic hyperactivity and reduce portal pressure while preserving the important splenic functions [40, 41]. A percutaneous approach through the femoral artery is feasible, and the procedure is short and minimally invasive, taking only one to two hours. It is effective in improving the liver function, decreasing variceal bleeding, treating hepatic encephalopathy, and improving blood cell counts in patients with portal hypertension [42]. A study of the acute effects of PSE on portal and visceral hemodynamics in cirrhotic patients reported that it produced an immediate portal decompression effect without decreasing portal perfusion and increased the blood flow in the hepatic artery and superior mesenteric vein, which was associated with an improved liver function [43]. Splenic abscess is an extremely serious complication, which may be triggered by post-embolization anaerobic bacterial growth, percutaneous contamination by exogenous bacteria, and retrograde infection of intestinal bacteria via portal reflux [44]. Unlike splenectomy, it is possible to selectively determine the extent of embolization. For cases of macro-splenomegaly or a poor hepatic function, it is also important to embolize in fractions from the beginning (keeping the embolization area at 30% per embolization) [40]. Since PSE has a poor immediate effect on stoma variceal bleeding, it needs to be combined with local hemostatic management [39]. After PSE in the present patient, the platelet count improved, and chemotherapy was able to be restarted. It is important to have a high index of suspicion for the diagnosis of hepatic SOS and to initiate treatment promptly. Patients with mild or moderate severity SOS generally do well with supportive care measures alone, but they must be monitored for progression to severe disease [8]. Defibrotide is a sodium salt of single-stranded oligodeoxyribonucleotides derived from DNA of the porcine intestinal mucosa [45] that was approved for the treatment of severe hepatic SOS after HCT in Japan in 2019. The mechanism of action is not well understood, but it may involve endothelial protection, restoration of the thrombo-fibrinolytic balance, and/or anti-inflammatory properties [46, 47]. Defibrotide treatment for severe SOS was associated with improved survival, according to a multicenter study of defibrotide treatment versus matched historical control patients [48], and a systematic review of 17 studies [49]. In Japan, defibrotide is currently approved only for use in severe SOS after HCT; however, the collection of further data on its use will be important because of concerns about the high incidence of SOS caused by oxaliplatin. Currently, we believe that it is better to follow patients with hepatosplenomegaly and collateral hyperplasia with attention to the risk of developing SOS under the use of oxaliplatin and to individualize the treatment as appropriate.

We searched for reports from PubMed (1950 to January 2022) using the keywords “stomal varices”, “stomal variceal”, or “stoma varix”. Among them, we extracted reports of stomal varices related to oxaliplatin-based chemotherapy in colorectal cancer cases and found six reports, including our case [5, 35, 44, 50, 51] (Table 2). There were five cases of liver metastasis, and five cases were diagnosed as SOS. All patients received oxaliplatin-based first-line chemotherapy, five patients received FOLFOX, and only our patient received CAPOX. The median cycle was 11.5 (5–13) courses. Two patients underwent variceal embolization and sclerotherapy via the percutaneous transhepatic approach. One case was treated with palliative ligation and percutaneous sclerotherapy, but rebleeding occurred, and PTO was performed later. None of them had rebleeding of stomal varices after PTO. Our patient underwent PSE, and one patient underwent TIPS for portal hypertension. The patient who underwent TIPS showed worsening of hepatic encephalopathy 6 weeks after the procedure. In our case, after direct embolization of stomal varices with PTO, PSE was safely performed to correct portal hypotension and thrombocytopenia. Five months have passed since PSE, and chemotherapy is ongoing without any recurrence of stoma bleeding.

**Table 2:** Reported cases of stomal varices after oxaliplatin-based chemotherapy

No.	Author (year)	Age	Sex	Location of CRC	Surgical treatment	Liver metastasis	SOS	Other liver condition	Chemotherapy	Cycle	L-OHP (mg/m^2^)	Onset of stomal varices	Treatment	Recurrent (time to recurrence)	Recurrent treatment	Treatment for portal hypertension	Outcome period (months)	Outcome	Cause of death
1	E Theophilidou (2012) [44]	64	M	R	Hartmann’s operation	+	+		FOLFOX	12	85	NA	Ligation/PS	+ (1 month)	PTO	None	6	Alive	
2	Sonoda H (2015) [5]	65	F	R	Sigmoid colostomy	+	+		mFOLFOX6	12	85	Unknown	TIPS	–	–	None	12	Death	Cancer death
3	MJ Maciel (2016) [35]	68	F	R	Palliative colostomy	+	NA		mFOLFOX6	5	85	4 cycles from start of chemotherapy	PTO	–	–	None	6	Alive	
4	Yamaguchi H (2018) [50]	27	F	T	Hartmann’s operation	+	+	Stenosis of RHV/IVC	mFOLFOX6 + Bev	13	85	6 mos. from start of chemotherapy	PE/PS	–	–	None	NA	Death	Liver failure
5	Uehara H (2020) [51]	66	F	R	Colostomy + ileum bypass → LAR → liver resection	+	+		mFOLFOX6 + Bev	9	85	4 years from end of chemotherapy	Open abdominal sclerotherapy	–	–	None	12	Alive	
6	Our case	46	M	R	APR	–	+		CAPOX + Bev	11	125	5 mos. from start of chemotherapy	PTO	–	–	PSE	7	Alive	

*M* male, *F* female, *CRC* colorectal cancer, *R* rectum, *T* transverse, *SOS* sinusoidal obstruction syndrome, *L-OHP* oxaliplatin, *APR* abdominoperineal resection, *LAR* low anterior resection, *NA* not available, *RHV* right hepatic vein, *IVC* inferior vena cava, *mos*. months, *PS* percutaneous sclerotherapy, *PSE* partial splenic embolization, *PTO* percutaneous transhepatic obliteration, *TIPS* transjugular intrahepatic portosystemic shunt, *PE* percutaneous embolization used

## Conclusion

We encountered a case of stomal varices due to portal hypertension caused by SOS after oxaliplatin administration that was treated by PTO and PSE. Considering the invasiveness and complications, PTO and PSE were the optimal choices. We suggest that staged treatment be considered an important treatment option for stomal varices and splenomegaly associated with SOS.
